# Clinical utility of chromosomal microarray analysis in the diagnosis and management of monosomy 7 mosaicism

**DOI:** 10.1186/s13039-014-0093-4

**Published:** 2014-12-04

**Authors:** Alka Chaubey nee Dwivedi, Michael J Lyons, Kat Kwiatkowski, Frank O Bartel, Michael J Friez, Kenton R Holden, Eric T Fung, Barbara R DuPont

**Affiliations:** Greenwood Genetic Center, Greenwood, SC USA; Affymetrix, Inc, Santa Clara, CA USA

**Keywords:** Monosomy 7, Mosaicism, Myelodysplasia, Microarray

## Abstract

There have been dramatic improvements in our ability to more accurately diagnose the underlying genetic causes of developmental delay/intellectual disability; however, there is less known about the treatment trajectory and whether or not patient management and outcomes have changed due to the information gained from genetic testing. Here we report a case study of a 20-month-old male first referred to the genetics clinic in 2008 for interhemispheric cysts, agenesis of the corpus callosum, left cortical dysplasia, and developmental delay of unknown etiology. The diagnostic work-up for this patient included chromosomal microarray which detected >20% mosaicism for monosomy 7, which raised concern for a possible myelodysplastic syndrome. The clone was not detected in stimulated peripheral blood cultures and his karyotype was reported as a normal male. Because of this microarray finding, he was referred to pediatric hematology/oncology where he was confirmed to have a pre-symptomatic diagnosis of myelodysplastic syndrome and was treated with chemotherapy and a bone-marrow transplant. This case illustrates the clinical utility of microarray testing and the importance of long-term follow-up to assess patient outcomes.

## Background

Over the past ten years, a dramatic shift in clinical laboratory testing has taken place in the assessment of patients with suspected genetic abnormalities related to developmental delay/intellectual disability, congenital anomalies, and dysmorphic features from low resolution techniques to high resolution genomic technologies.

Traditionally, karyotyping and fluorescence *in situ* hybridization (FISH) were the methods used for cytogenetic analysis. Karyotyping can detect aberrations at a resolution averaging 5–10 megabases while FISH, a more sensitive technique, enables visualization of targeted chromosomal regions at a resolution of greater than or equal to 150 kilobases. However, FISH is limited in its application by the probes used to detect a specific genetic locus. For this reason, karyotyping and FISH have been commonly used in tandem in clinical practice (karyotyping to visualize the whole genome and FISH to target specific locations at a higher resolution) in order to improve diagnostic yield.

Over the past decade, microarray technology has transitioned from the research laboratory to the clinical laboratory and is used for assessing copy number variations (CNVs) in patients undergoing diagnostic testing for developmental delay/intellectual disability due to its ability to assess DNA copy number across the entire genome at a resolution not possible with karyotype or FISH [1]. Furthermore, the American College of Medical Genetics and Genomics (ACMG) Practice Guidelines published in 2011 and revised in 2013 recommend that genomic microarrays be used as first-tier tests for the postnatal evaluation of individuals with developmental delay/intellectual disability [2,3]. However, current laboratory practices for chromosomal testing vary and can range from a microarray as the sole diagnostic test of choice, to a combination of several methods (e.g. microarray, karyotype, FISH, and Fragile X testing by polymerase chain reaction based methods) [4,5].

There are many reports in the scientific literature about the increase in diagnostic yield by employing microarray technology [1]. However, there is less information about how this improved diagnostic yield translates into better clinical outcomes. The current case serves as an example of this enhanced patient outcome made possible by chromosomal microarray. Secondly, this also highlights that improvements in diagnostic yield are not limited only to increased resolution but also allow for the detection of chromosome abnormalities in non-dividing cells.

## Case presentation

A prenatal ultrasound at 22 weeks gestation revealed agenesis of the corpus callosum, suspected interhemispheric cysts, and cortical dysplasia for the male patient. There were no other complications or exposures during the pregnancy. The delivery was normal. The birth weight was 3,976 grams (75-90^th^ percentile), the birth length was 53.5 cm (75-90^th^ percentile), and the birth head circumference was 36.5 cm (75-90^th^ percentile). He was noted to be irritable at birth, but was otherwise well. The child’s parents are both healthy. The mother previously had a miscarriage at 12 weeks. The child has 1 healthy brother.

**Figure 1:**
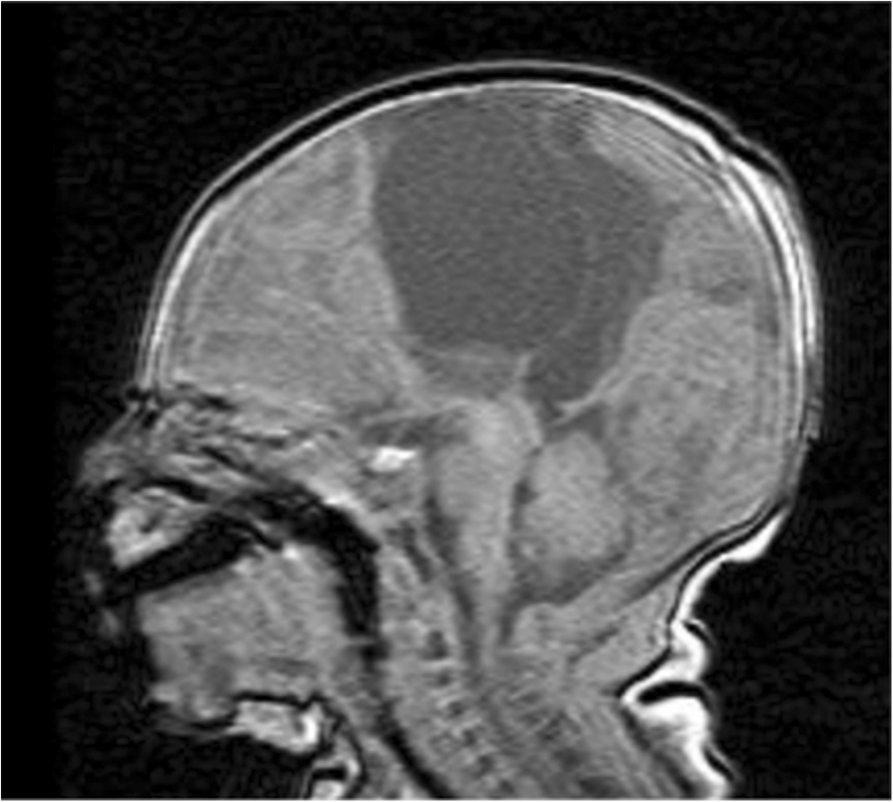
**Sagittal T1 image at 5 days old demonstrating interhemispheric cysts and agenesis of corpus callosum.**

A brain MRI at 5 days old confirmed agenesis of the corpus callosum, two expanding interhemispheric cysts, and left focal cortical dysplasia (Figure 1). At 1 month of age, the patient underwent a left parietal craniotomy and fenestration of the interhemispheric cysts. Post-surgery, a pediatric head ultrasound revealed possible postoperative hematoma. At 2 months of age, a head ultrasound revealed a slight decrease in size of the multiloculated midline cysts and a cystic change within the parasagittal intraparenchymal hematoma compatible with encephalomalacia. A brain MRI at 7 months confirmed the presence of two interhemispheric cysts and agenesis of the corpus callosum along with left frontal encephalomalacia and left frontal cortical dysplasia. The patient underwent another craniotomy and fenestration of the re-accumulated cysts at 8 months. He developed aseptic meningitis following surgery. At 13 months of age, the patient received a shunt for decompression of the interhemispheric cysts with good results. The patient had developed right hemiparesis which improved after the shunt placement.

The patient had a number of normal abdominal ultrasounds. He had chronic ear infections and received PE tubes at 16 months old. Developmentally, the patient sat at 6–7 months and walked at 19 months. He was referred to speech therapy due to concerns about speech delay.

**Figure 2:**
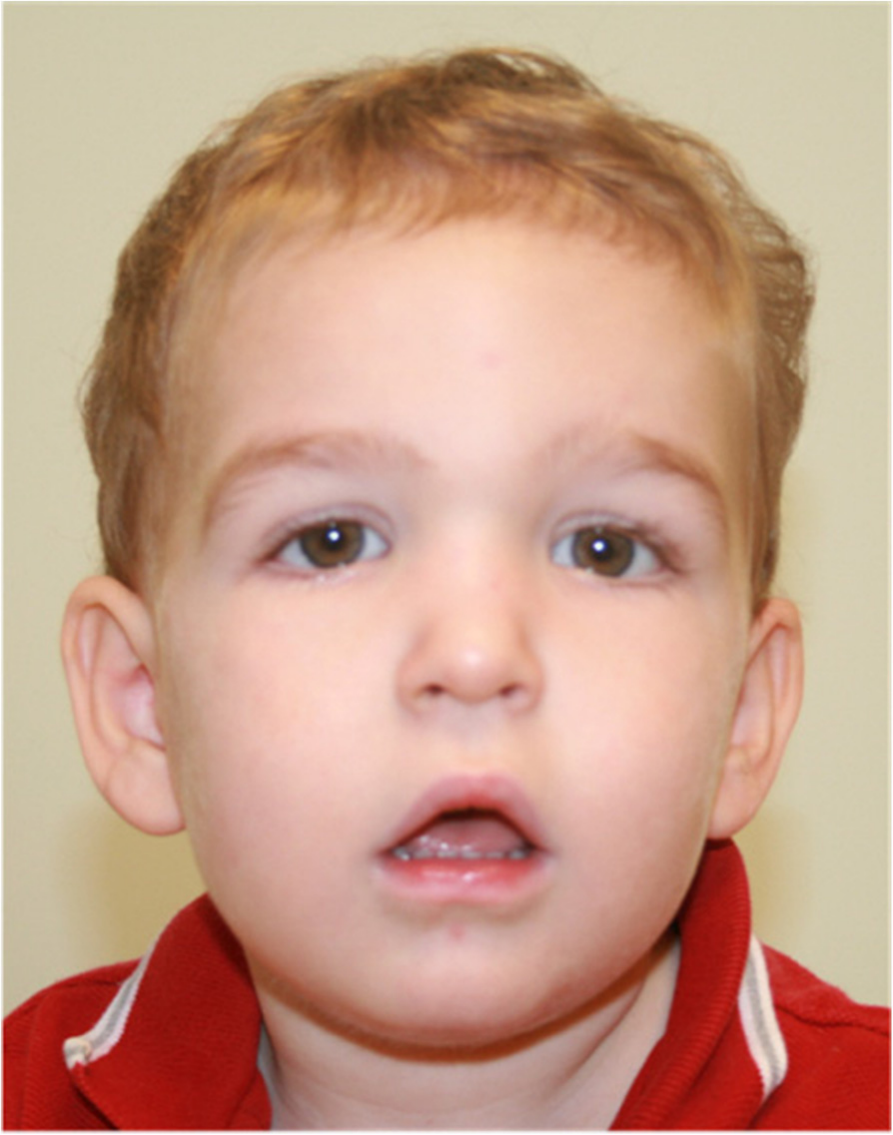
**Facial features at 20 months old demonstrating down-slanting palpebral fissures with full eyebrows.**

The patient was first referred to the genetics clinic at 20 months of age due to the previously described brain abnormalities and developmental delay of unknown etiology. He had a length of 80.5 cm (10^th^ to 25^th^ percentile), a weight of 14.1 kg (90^th^-95^th^ percentile) and a head circumference of 50.3 cm (95^th^ percentile). He was alert and interactive. He periodically pointed and grunted. He said “mama” and “dada” nonspecifically. He had slightly down-slanting palpebral fissures with full eyebrows, mildly increased tone on the ride side, and a wide-based, unsteady gait (Figure 2).

### Materials and methods

**Figure 3:**
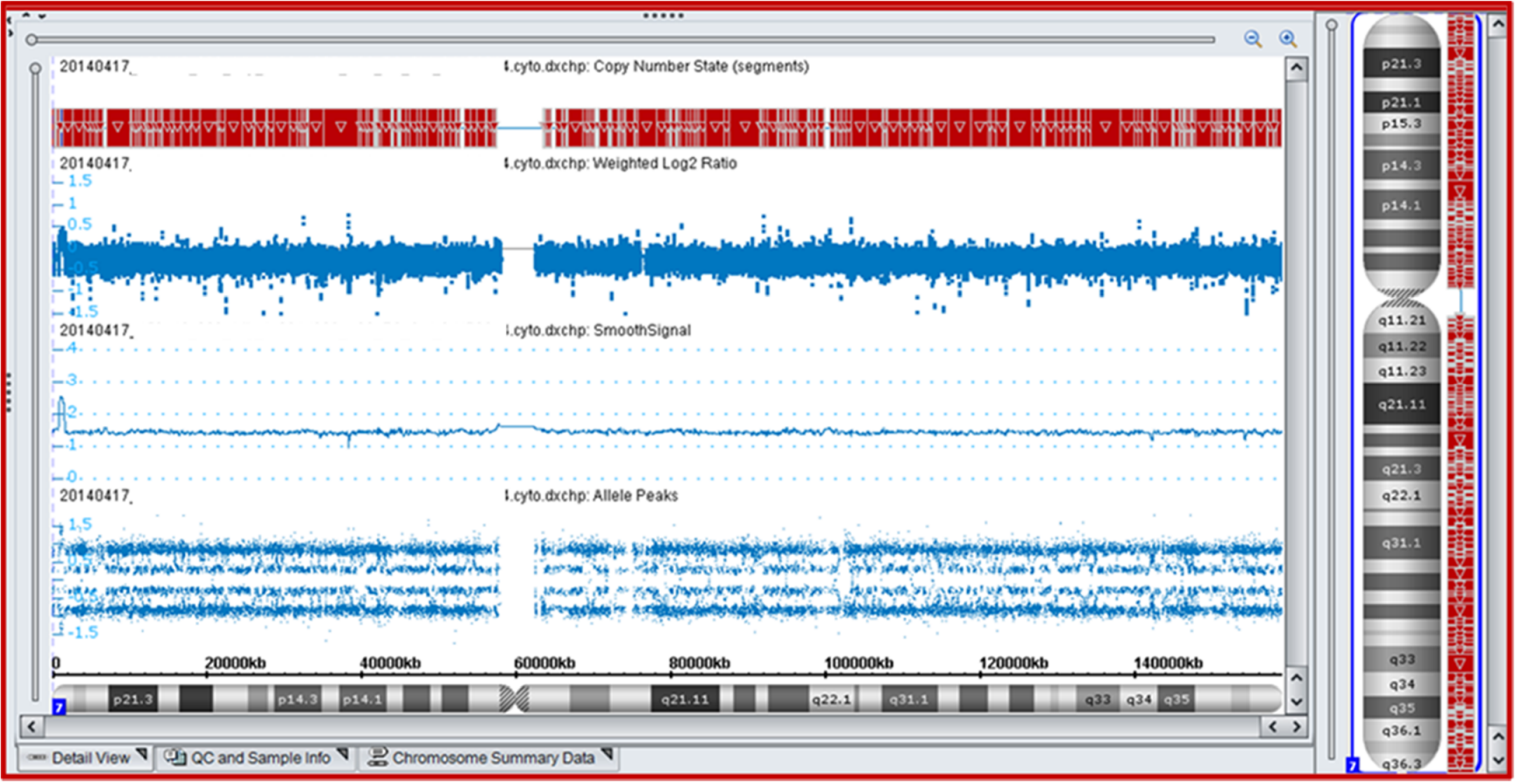
**Chromosomal microarray analysis showing mosaic monosomy 7 using the CytoScan Dx Assay.** The smooth signal plot (non-polymorphic probes) shows the mosaic loss represented as the line between copy states 1 and 2. The four tracks on the allele plots (SNP probes) also confirm the copy number probes and reflect the ~50% mosaic loss of chromosome 7.

In order to provide an explanation for his clinical features, high-resolution chromosome analysis (karyotype) and chromosomal microarray analysis were requested. Chromosomal microarray analysis was performed on 4 platforms using the 105 K Syndrome Plus (Oxford Gene Technology, UK), Genome-wide SNP 6.0 microarray, CytoScan HD array and CytoScan Dx Assay (Affymetrix, Inc. USA, Figure 3) as per manufacturers’ instructions.

### Results

Chromosome analysis revealed a normal, 46,XY karyotype. Initially, neither microarray nor MLPA subtelomere analysis could be performed on the original blood samples (taken at birth) due to DNA degradation.

**Figure 4:**
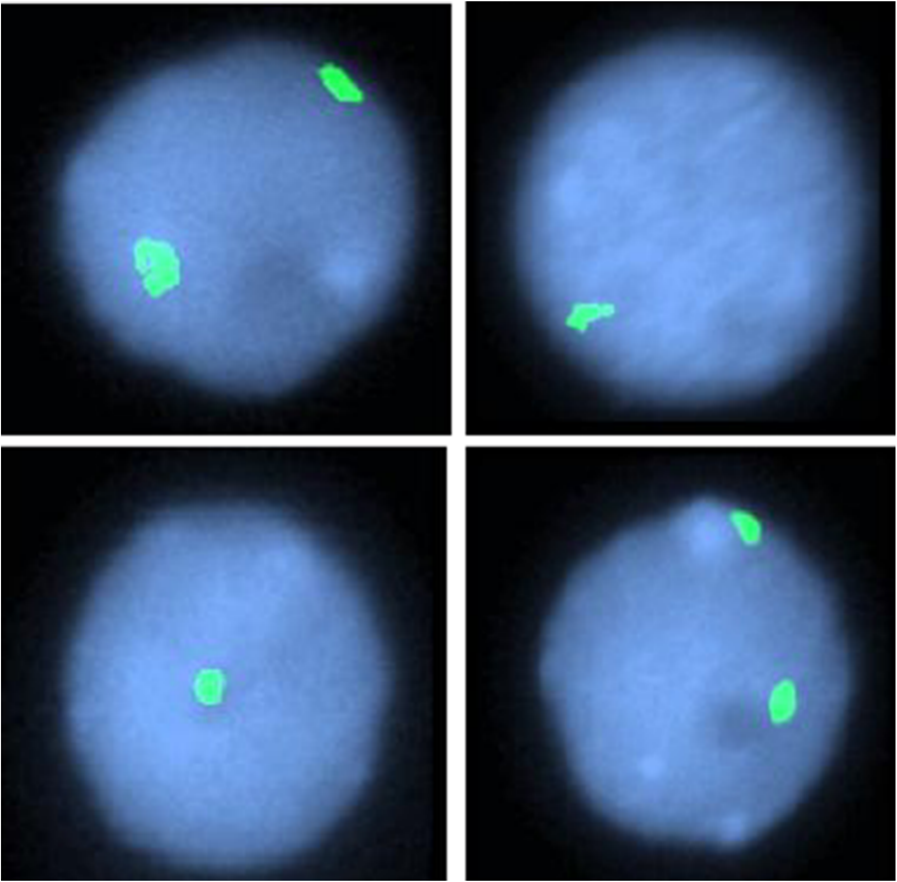
**Fluorescence in situ hybridization using the CEP 7 probe performed on the direct peripheral blood specimen of the proband showing normal (2 green signals) and abnormal (1 green signal) interphase nuclei.**

When the patient was 2 years old, microarray analysis was repeated and revealed mosaicism (>20%) for monosomy 7 (Figure 3). FISH analyses were performed on both the unstimulated and stimulated cultures on interphase cells using the CEP 7 (D7Z1) Alpha Satellite Probe specific for 7p11.1-q11.1 (Vysis #32-130007). Mosaic monosomy 7 was identified in only 37 of 714 cells (~5%) in the stimulated cultures and 21 of 100 (~21%) cells in the unstimulated cultures (Figure 4). An attempt was made to perform FISH analysis on the stimulated culture cell pellet (from which the karyotypes were obtained) but failed to identify any metaphases with monosomy 7 which most likely indicates the subpopulation of cells stimulated by PHA does not contain −7 cells.

Monosomy 7 mosaicism has been causally linked to gain of function mutations in RAS pathway genes [6]. As a result, clinical sequencing of the RAS pathway genes *PTPN11*, *SOS1*, *RAF1*, and *KRAS* was completed and all results were normal.

Monosomy 7 mosaicism was not felt to be the explanation for the patient’s clinical features; however, due to reports of an association between monosomy 7 mosaicism and myelodysplasia, the patient was referred to a pediatric hematologist/oncologist for further evaluation. The patient had a bone marrow biopsy which revealed evidence of myelodysplasia. He subsequently had chemotherapy and a bone marrow transplant at 4 years old.

Almost one year after surgery, he was seen at the genetics clinic for a follow-up visit. It was noted that several follow-up bone marrow biopsies indicated normal bone marrow without evidence of myelodysplasia. Chromosome analysis and FISH for monosomy 7 were normal following the bone marrow transplant. The patient was doing well with no subsequent issues and was not taking any medications. He had a number of dental cavities attributed to the use of chemotherapy but was making developmental progress. He had decreased use of his right hand and right leg and preferred to use his left hand.

### Discussion

Monosomy 7 is one of the most frequent chromosomal abnormalities observed in myelodysplasia and acute myelogenous leukemia (AML) [7,8]. Other congenital bone marrow disorders, such as Fanconi anemia, congenital neutropenia and familial monosomy 7, predispose to leukemia usually through a myelodysplastic phase [5,9-11]. Early detection and identification of monosomy 7 by microarray may allow for earlier diagnosis and intervention which may translate into improved patient prognosis.

We report the detection of monosomy 7 mosaicism in a child referred for genetic testing due to mild developmental delay, agenesis of the corpus callosum, interhemispheric cysts, and left cortical dysplasia. The percent mosaicism for monosomy 7 was readily detected and quantified by microarray and FISH analysis but not by high resolution chromosome analysis.

Monosomy 7 mosaicism was not felt to be the explanation for his presenting clinical features. Neither did he have symptoms, such as easy bruising or petechiae that would suggest an underlying diagnosis of myelodysplasia. Due to the association of monosomy 7 mosaicism with myelodysplasia, he was referred to hematology/oncology and confirmed to have myelodysplasia based on bone marrow biopsy. He subsequently had a bone marrow transplant with apparently curative outcome.

Monosomy 7 is considered a poor prognostic sign in individuals with myelodysplasia [10]. However, the early detection of monosomy 7 prior to the development of any related hematologic symptoms is believed to have improved our patient’s long-term prognosis. If microarray had not detected monosomy 7 mosaicism during his evaluation for other unrelated clinical features, the diagnosis of myelodysplasia and subsequent bone marrow transplant would likely have been significantly delayed, resulting in a worse prognosis for our patient. Given the lack of correlation of the level of mosaicism and myelodysplaisa, any level of mosaicism for monosomy 7 would warrant further hematological evaluation for possible myelodysplasia.

## Conclusion

In conclusion, we believe that the fortuitous identification of monosomy 7 mosaicism by microarray significantly improved the clinical outcome for our patient. The microarray result led to a referral to hematology/oncology which would not have otherwise happened until he developed symptoms of a hematologic disorder. This demonstrates the benefits of multi-disciplinary collaboration when a significant microarray result is identified as having a potential impact on the management of a patient.

## Consent

We would like to thank the family for their participation and written informed consent was obtained for the publication of this case report and any accompanying images. We also thank Dr. Michelle Hudspeth for the care provided and follow-up clinical reports after identification of myelodysplasia in the patient.
